# Trends in the Prevalence and Antibiotic Resistance of Non-tuberculous Mycobacteria in Mainland China, 2000–2019: Systematic Review and Meta-Analysis

**DOI:** 10.3389/fpubh.2020.00295

**Published:** 2020-07-28

**Authors:** Lei Zhou, Da Xu, Hancan Liu, Kanglin Wan, Ruibai Wang, Zaichang Yang

**Affiliations:** ^1^College of Pharmacy, Guizhou University, Guiyang, China; ^2^State Key Laboratory for Infectious Disease Prevention and Control, Chinese Centre for Disease Control and Prevention, National Institute for Communicable Disease Control and Prevention, Beijing, China; ^3^Guangdong Key Laboratory for Diagnosis and Treatment of Emerging Infectious Diseases, Shenzhen Third People's Hospital, Southern University of Science and Technology, Shenzhen, China

**Keywords:** non-tuberculous mycobacteria, atypical mycobacteria, prevalence, antibiotic resistance, dominant species, geographic distribution

## Abstract

**Background:** China is a high-burden country of tuberculosis. The proportion of diseases caused by non-tuberculous mycobacteria (NTM) has increased, seriously affecting the prevention, control, and management of tuberculosis (TB) and posing a significant threat to human health. However, there is a lack of an organized monitoring system for NTM such as that used for tuberculosis. Comprehensive data on patient susceptibility, dominant species, and drug resistance profiles are needed to improve the treatment protocols and the management of NTM.

**Methods:** Primary research reports of NTM clinical specimens from mainland China published between January 1, 2000 and May 31, 2019 were retrieved from four online resources (BIOSIS, Embase, PubMed, and Web of Science) and three Chinese medical literature databases (CNKI, Wanfang, and Vip) as the Preferred Reporting Items for Systematic Reviews and Meta-Analyses.

**Results:** In total, 339 publications were included in the systematic review, 129 were used in the drug susceptibility analysis, and 95 were used in the meta-analysis. Traditional culture using Lowenstein–Jensen slants combined with P-nitrobenzene acid and thiophene-2-carboxylic acid hydrazine differential medium and proportional method was most commonly used for the isolation, identification, and drug susceptibility testing of NTM in China. The crude isolation rate for NTM among TB suspected cases was 4.66–5.78%, while the proportion of NTM among *Mycobacterium* isolates was 11.57%. *Mycobacterium abscessus* and *Mycobacterium avium* complex were the most common clinical NTM species. NTM only showed general sensitivity to ethambutol, linezolid, clofazimine, amikacin, tobramycin, and clarithromycin.

**Conclusions:** The prevalence of NTM in China has shown a decreasing trend. *M. abscessus* was replaced as the dominant species by *Mycobacterium intracellulare* over the course of the study. The geographic diversity of different species showed the effects of environmental and economic factors on the distribution of NTM and indicated that there were important factors still not identified. While there were only a limited number of antibiotics to which NTM showed any sensitivity, the drug resistance profiles of the isolates were highly variable and thus more caution should be taken when empirically treating NTM infection.

## Introduction

Non-tuberculous mycobacteria (NTM) species spread among five genera (*Mycobacterium, Mycobacteroides, Mycolicibacillus, Mycolicibacter*, and *Mycolicibacterium*) of the family *Mycobacteriaceae* (1). To date, more than 250 taxa (species and subspecies) have been identified as NTM and are ubiquitously distributed throughout aquatic and terrestrial environments. Although most NTM species are non-pathogenic and rarely observed in clinical samples, up to 60 NTM species have repeatedly been recovered from clinical specimens and proved to cause human diseases (2, 3).

The incidence and the disease burden of NTM infection have increased significantly worldwide as a result of changes in demographics, advances in radiological diagnosis of pulmonary abnormalities and identification techniques of *Mycobacterium* species, and the control and decline of tuberculosis (TB). The prevalence of NTM even exceeded *Mycobacterium tuberculosis*, the causative agent of TB, in the United States (4). Moreover, among suspected multidrug-resistant TB patients who did not respond to 2–3 months of treatment with first-line anti-TB drugs, about 30% were actually NTM infection (5), with the actual NTM infection rates likely to be higher. Patients with impaired immunity as a result of malignancies, organ transplantation, and HIV infection, those with chronic pulmonary diseases, and the elderly are at a higher risk of NTM infection (4, 6, 7). NTM co-infection with HIV and *M. tuberculosis* has negatively impact on TB management.

Because NTM infection is not a legally required notifiable disease, coupled with the cumbersome operation of NTM identification methods, there is not such a sophisticated and organized TB monitoring system for NTM in China, the same as is the case in Europe and most other countries (8). An epidemiological analysis of NTM depends mainly on systematic review and meta-analysis. Because NTM are a large heterogeneous group of microorganisms, patient susceptibility, dominant species, and drug sensitivity, including anti-tuberculosis drugs, vary greatly within species. However, such information has significant reference value for the treatment and the management of these infections, especially in low- and middle-income countries without sufficient resources or laboratory equipment to conduct the clinical tests. Thus, in the current study, we systematically reviewed and analyzed reports on NTM from mainland China, published within the last 20 years, to explore the epidemic characteristics and trends in NTM prevalence, distribution, and drug resistance in China.

## Materials and Methods

### Search Strategy and Inclusion and Exclusion Criteria

The study was carried out according to the Preferred Reporting Items for Systematic Reviews and Meta-Analyses. All studies involving NTM clinical specimens from mainland China and published between January 1, 2000 and May 31, 2019 were retrieved from four online resources (BIOSIS, Embase, PubMed, and Web of Science) and three Chinese medical literature databases (CNKI, Wanfang, and Vip). The screening terms included the combination of “China/Chinese” and medical subheadings (MeSH) or key words (NTM, atypical mycobacterium, atypical mycobacterial infection, non-tuberculous, non-tuberculous mycobacterium, non-tuberculosis, non-tuberculous, as well as the names of the 249 NTM species and subspecies reported to date). Additional studies were added by retrospective searches.

Only original articles were included in the analysis. The inclusion criteria required that the studies clearly described the NTM isolation and identification methods used. The samples should be from patients in mainland China, with a definite sample size and number of NTM isolated. For reports on drug sensitivity, standardized methods of drug sensitivity testing (DST) should be used and breakpoints for drug resistance determination should be noted. Among duplicate studies from the same source conducted at the same location and time, only the study with the largest sample size was included. For studies using NTM isolates collected from imported patients, the environment or animal was excluded. Review articles and conference abstracts without the full text were also excluded.

### Data Extraction and Management

The following data were extracted from the included full-text publications: sample source, patient characteristics, collection time, location, methods of identification/culture and DST, number of cases and strains isolated (NTM and total mycobacteria, if applicable), NTM species, breakpoints for drug resistance determination, drug resistance rates, authors, and affiliations. Two researchers independently performed the literature search and data entry. Inconsistencies were rechecked to obtain consensus.

### Data Analysis

Stata 14 (9) and NoteExpress 3.0 (AEGEAN, Beijing, China) were used for data management and meta-analysis. Heterogeneity was examined using the *I*^2^ statistic. Funnel plots were used to quantitatively assess publication bias. The pooled prevalence of NTM and each species with corresponding 95% confidence intervals (CIs) was calculated using forest plots in a random-effect model. Stratified statistical analyses were subsequently performed with respect to species, isolation time, and geographic area.

## Results

### Screening Results and Characteristics of the Included Studies

**Figure 1:**
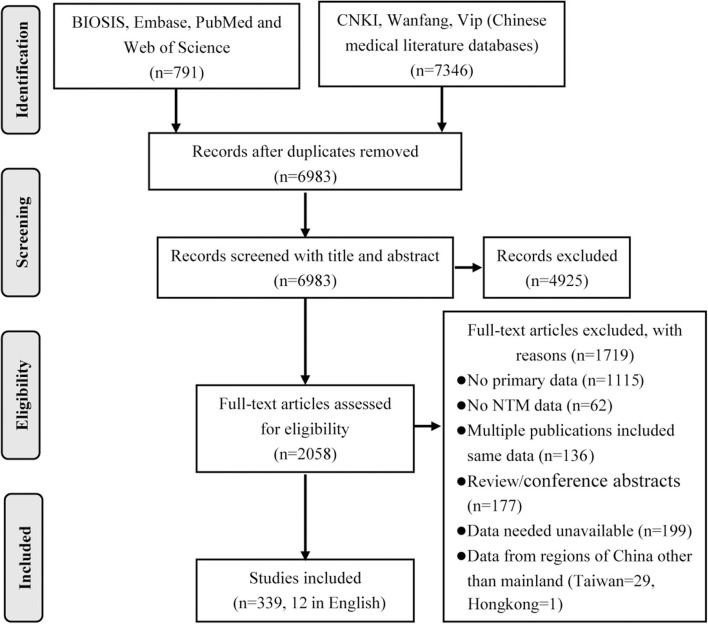
Preferred Reporting Items for Systematic Reviews and Meta-Analyses flow diagram.

A total of 8,137 publications were obtained by database search using the combination terms set for this study. After eliminating duplicates and irrelevant studies based on the title and abstract evaluations, 2,058 publications were carried forward for full-text evaluation. As a result, 1,719 papers were further excluded, leaving 339 papers for inclusion in the systematic review, including 12 in English and 327 in Chinese (Figure 1).

The publications used for the analyses included 55 case reports, 141 studies with <50 isolates, 70 with between 50 and 100 isolates, and 73 with >100 isolates. Geographically, the included studies covered 29 of the 31 provinces of mainland China. In total, 296 publications were reports from a single hospital, 43 covered multiple hospitals/centers, and 19 included samples from multiple provinces. Among the papers, 136 described single-year studies and 203 were multi-year studies, of which 36 contained data from >5 years. In addition, 67 publications involved only one NTM species, while 170 publications included species composition information. Of the 339 papers, 139 publications also included DST data.

### NTM Culture and Identification Methods

Traditional culture on Lowenstein–Jensen (L–J) slants combined with P-nitrobenzene acid and thiophene-2-carboxylic acid hydrazine differential medium was the most commonly used isolation and identification method for NTM over the 20-year study period in mainland China (Supplementary Tables 1, 2). The use of commercial BACTEC™ medium (MGIT™ 960, 460, 9120, and FX40) and MacC agar increased in 2010, with use of the latter medium almost surpassing that of the L–J slants for NTM culture over the past 5 years. Molecular detection methods, particularly polymerase chain reaction (PCR)-based analysis and sequencing of conserved genes, have been applied to NTM species identification since 2005. Among 87 studies (9,628 NTM strains) using the PCR-sequencing identification method, 57 (7,284 NTM strains) conducted multi-locus analysis and 30 (2,344 NTM strains) used single-gene sequencing. Among molecular studies, 16S rRNA, *hsp65*, and *rpoB* were the three most commonly used target genes.

### NTM Prevalence and Species Recovered From Clinical Specimens

**Figure 2:**
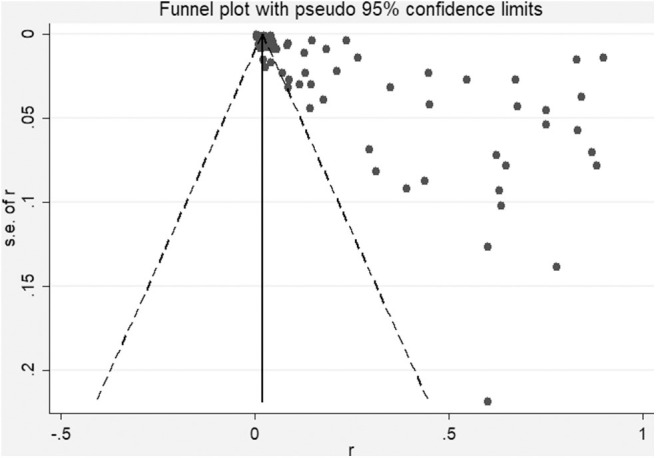
Heterogeneity among the included studies. Dots represent individual studies.

Over the 20-year period from 2000 to 2019, 13,653 NTM isolates were recovered from 293,011 clinical suspected TB samples (patients with TB signs or symptoms, including pulmonary and extrapulmonary TB) in mainland China. In these publications, 58 papers also reported the total isolations of mycobacteria, which was 8,973 NTM among 77,964 mycobacteria from 219,829 samples. Thus, the crude isolation rate for NTM of TB suspects was 4.66%, with the proportion of NTM among *Mycobacterium* isolates at 11.57% in China. Excluding case reports, there was significant heterogeneity among the studies included in the meta-analysis (*X*^2^ = 17,579.19, *I*^2^ = 99.5%, *P* < 0.001). After excluding the studies with <100 NTM isolates, the isolation rate for NTM increased to nearly 5.78%. However, even if only studies with >200 NTM isolates were included, the heterogeneity remained statistically significant. Therefore, a random-effect model was used for the meta-analysis (Table 1 and Figure 2). In addition, stratified statistical analyses were conducted. From 2000 to 2019, the 5-year isolation rates for NTM in TB suspects were 3.01 (2.67–3.36), 6.71 (6.22–7.20), 3.01 (2.84–3.17), and 7.91% (7.32–8.49%), respectively. The proportion of NTM among the *Mycobacterium* isolates were 8.15, 22.03, 8.08, and 18.20%, respectively, but there was no significant difference between the two highest proportions (*X*^2^ = 5.97, *P* > 0.001). In the three economic zones of China, coastal, central, and western, the separation rate of NTM was higher in coastal areas than in inland areas, which showed a statistical geographic difference.

**Table 1:** Non-tuberculous mycobacteria (NTM) isolation rates in mainland China and heterogeneity among studies included in the meta-analysis.

	**Classification**	**Number of publications**	**Isolation rate (95% CI)**	**Proportion of NTM in *Mycobacterium* (%)**	**Heterogeneity**		
					**χ^**2**^**	***I*^**2**^**	***P***
Overall effect		95	4.66 (4.58–4.74)	11.57	17,579.19	99.5	<0.001
NTM isolation	100≤	26	5.33 (5.23–5.42)	13.10	13,709.38	99.8	<0.001
	200≤	17	5.78 (5.67–5.88)	14.18	12,413.19	99.9	<0.001
Region^a^	Coastal	62	4.68 (4.60–4.76)	12.40	11,902.22	99.5	*P* <0.001
	Central	27	4.04 (3.76–4.33)	6.13	3,121.66	99.2	*P* <0.001
	Western	14	2.90 (2.70–3.10)	6.98	1,514.36	99.1	*P* <0.001

a *The regions were divided according to the economic belt of China. The coastal provinces on the mainland include Shandong, Hebei, Liaoning, Jiangsu, Tianjin, Zhejiang, Fujian, Shanghai, Guangdong, Hainan, and Guangxi; the central provinces include Beijing, Shanxi, Henan, Anhui, Hubei, Jiangxi, Hunan, Heilongjiang, and Jilin; the western provinces include Sichuan, Yunnan, Guizhou, Tibet, Chongqing, Shaanxi, Gansu, Qinghai, Xinjiang, Ningxia, and Guangxi Inner Mongolia*.

**Figure 3:**
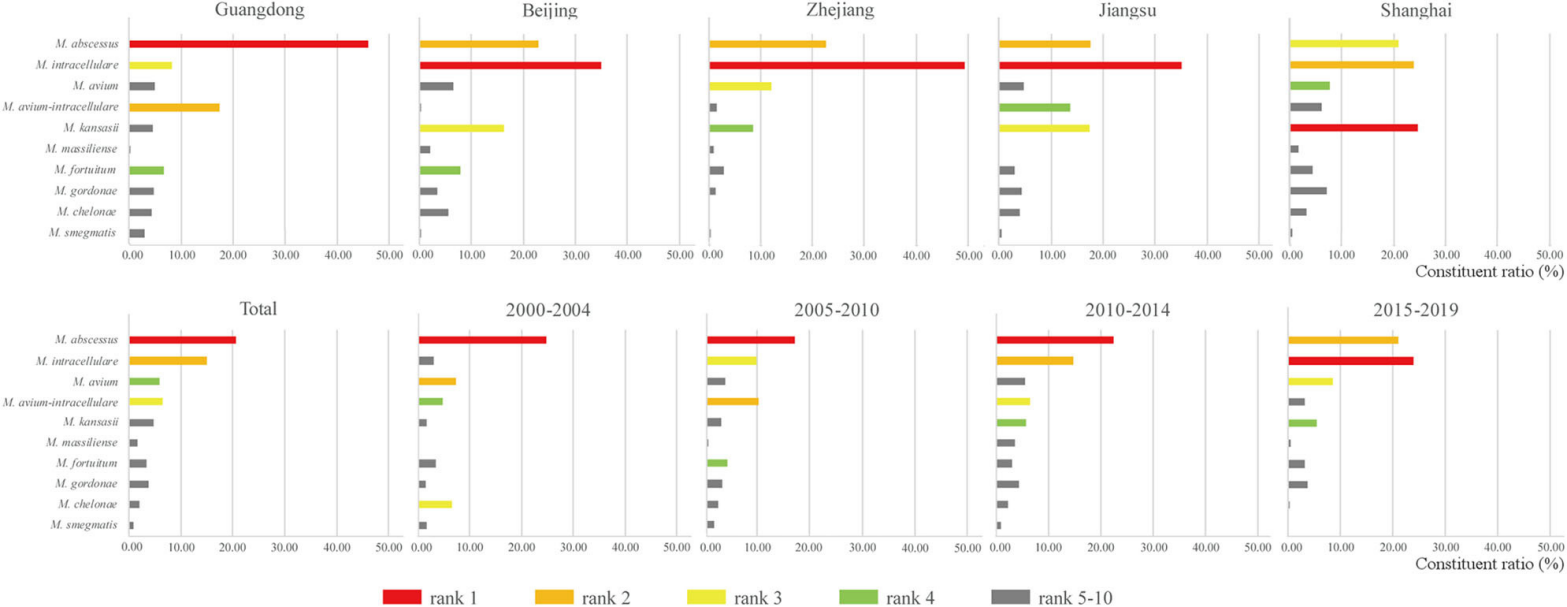
Most common clinical non-tuberculous mycobacteria (NTM) species in the five provinces with the highest NTM isolation and the four 5-year periods.

Overall, 26,407 NTM isolates from 237 studies were identified as belonging to 64 NTM species. Twenty-eight species had <10 isolates, 16 species had 10–50 isolates, five species had 50–100 isolates, and 15 species had >100 isolates. *Mycobacterium abscessus* and species belonging to the *Mycobacterium avium* complex (MAC) were always among the top three most prevalent species in the 20-year period (Figure 3 and Supplementary Table 3). At the species level, *M. abscessus* was the most prevalent NTM species in clinical samples from China prior to 2015. After 2015, even though the isolates decreased compared with the previous 5 years, *M. intracellulare* became the most prevalent NTM species after a significant decrease in the prevalence of *M. abscessus*. If analyzed at the species group (*M. abscessus*/*chelonae* and MAC) level, MAC were the dominant NTM species in China across the 20-year study period. In addition, the prevalence of *Mycobacterium chelonae* decreased, while that of *Mycobacterium kansasii* significantly increased. The largest numbers of NTM isolates were recovered from Guangdong, Zhejiang, Shanghai, Beijing, and Jiangsu provinces (Figure 3 and Supplementary Table 3). Interestingly, the proportions of the 10 most dominant NTM species differed among these provinces, with differences even observed among the three neighboring provinces, Zhejiang, Jiangsu, and Shanghai.

### Pre-existing Conditions and Clinical Symptoms of NTM Patients

A total of 70 papers and 1,689 patients were included in the analysis of the pre-existing conditions of NTM infections. The most common pre-existing conditions were HIV infection (41.33%), bronchiectasis (16.87%), previous pulmonary tuberculosis (15.87%), chronic obstructive pulmonary disease (11.37%), diabetes (5.33%), pleurisy (3.14%), hypoproteinemia (2.66%), anemia (1.84%), and hypertension (1.60%). The main risk factors for NTM in China were different from those reported in Europe (chronic obstructive pulmonary disease, bronchiectasis, and asthma) and in the United States (fibrocavitary disease and consolidating infiltrates) (10).

From 449 HIV co-infection patients, a total of 26 NTM species were recovered. In addition, four co-infection cases were positive for two different NTM species. NTM can cause bacteremia as well as skin/soft tissue, bone, joint, and eye infections, with different NTM species more commonly associated with different clinical manifestations. Only one species, *M. abscessus*, was reported in all reports of eye infection (Table 2).

**Table 2:** Syndromes caused by non-tuberculous mycobacteria (NTM) infection and related species.

	**HIV co-infection**	**Bacteremia**	**Pulmonary infection**	**Skin/soft tissue infection**	**Bone and joint infection**	**Eye infection**
NTM species in the top five	*M. avium*	*M. marinum*	*M. avium*	*M. chelonae*	*M. fortuitum*	*M. abscessus*
	*M. gordonae*	*M. abscessus*	*M. abscessus*	*M. marinum*	*M. abscessus*	
	*M. kansasii*	*M. avium*	*M. chelonae*	*M. fortuitum*	*M. marinum*	
	*M. chelonae*	*M. kansasii*	*M. intracellulare*	*M. abscessus*	*M. gordonae*	
	*M. abscessus*	*M. intracellulare*	*M. kansasii*	*M. avium*	*M. intracellulare*	
Case reported	449	49	2,417	1,090	9	25
Total NTM species identified	26	6	15	20	8	1
Number of cases of mixed infection of two or more NTMs	4	1	8	12		2

### DST Methods and Antibiotic Resistance of Chinese NTM Isolates

Overall, six DST methods were used in the included studies. The numbers of publications and NTM strains tested using each method are summarized by time period in Table 3, and the breakpoints used are summarized in Supplementary Table 4. The results showed that, since 2010, an increasing number of studies have assessed the antibiotic sensitivity of NTM. The gold standard DST method for mycobacterium, proportional method, and the traditional absolute concentration method were the most common testing methods used among these studies. However, the microporous plate method, which is easier to conduct and can obtain minimum inhibitory concentration values, has been used more frequently since 2015.

**Table 3:** The drug sensitivity testing methods used for non-tuberculous mycobacteria (NTM) in China.

**Method**	**Numbers of publication and NTM strains (ranks)**				**Total**
	**2000–2004**	**2005–2009**	**2010–2014**	**2015–2019**	
Proportion method	5 (1)	3 (2)	23 (2)	23 (1)	54 (1)
Absolute concentration method	2 (2)	9 (1)	25 (1)	11 (2)	47 (2)
Turbidimetry method	1 (3)	1 (4)	3 (3)	7 (4)	12 (3)
Alarmar blue method				10 (3)	10 (4)
K-B test	2 (2)	1 (4)	1 (4)		4 (5)
E-test		2 (3)			2 (6)
Total	10	16	52	51	129

**Figure 4:**
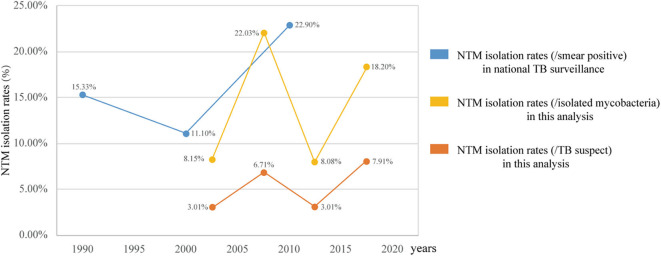
Antibiotic resistance rates of the top 10 clinical non-tuberculous mycobacteria species. The red lines indicate the average rates, while the black dots indicate the actual resistance rates reported in the papers. Inh, isoniazid; Rif, rifampicin; Emb, ethambutol; Lfx, levofloxacin; Mxf, moxifloxacin; Lzd, linezolid; Cfz, clofazimine; Cs, cycloserine; Imp, imipenem; Mpm, meropenem; Am, amikacin; Str, Streptomycin; Pto, protionamide; PAS, aminosalicylic acid; Cm, capreomycin; Km, kanamycin; Tbm, tobramycin; Smz, sulfamethoxazole; Pa, pasinizid; Rap, rifapentine; Rfb, rifabutin; Cfx, cefoxitin; Ofx, ofloxacin; Cip, ciprofloxacin; Gat, gatifloxacin; Azi, azithromycin; Clar, clarithromycin.

Although NTM are generally resistant to first-line anti-TB drugs, they are usually included in NTM DST. In the included publications, 65 NTM species were screened using a total of 41 different drugs. Among the 10 most common clinical NTM species, up to 27 drugs were tested against >50 NTM isolates from a single species across 141 studies. An analysis of antibiotic resistances rates among the 10 most prevalent NTM species (Figure 4 and Supplementary Table 5) showed that there were very few drugs to which the NTM isolates showed a high degree of sensitivity. Among the most common clinical species, *M. avium* showed the lowest levels of drug sensitivity (26.9%, 7/26, average drug resistance rate <50%), followed by *M. abscessus* (33.3%, 9/27), *M. avium-intracellulare* (37.5%, 6/16), *M. fortuitum* (37.5%, 9/24), *M. chelonae* (37.5%, 9/24), and *M. intracellulare* (38.5%, 10/26). Only *M. kansasii* and *Mycobacterium gordonae* were sensitive to more than half of the tested drugs, at 52% (13/25) and 66.7% (16/24), respectively. Among the first-line anti-TB drugs, almost all NTM species were highly resistant to isoniazid. MAC, *M. kansasii*, and *M. gordonae* were moderately sensitive to rifampicin. Ethambutol was relatively the most sensitive first-line anti-TB drug for NTM. Among other drugs, NTM only showed general sensitivity to linezolid (group A drug), clofazimine (group B drug), amikacin (group C drug), tobramycin, and clarithromycin, which could be used as clinically recommended drugs for the treatment of NTM.

## Discussion

**Figure 5:**
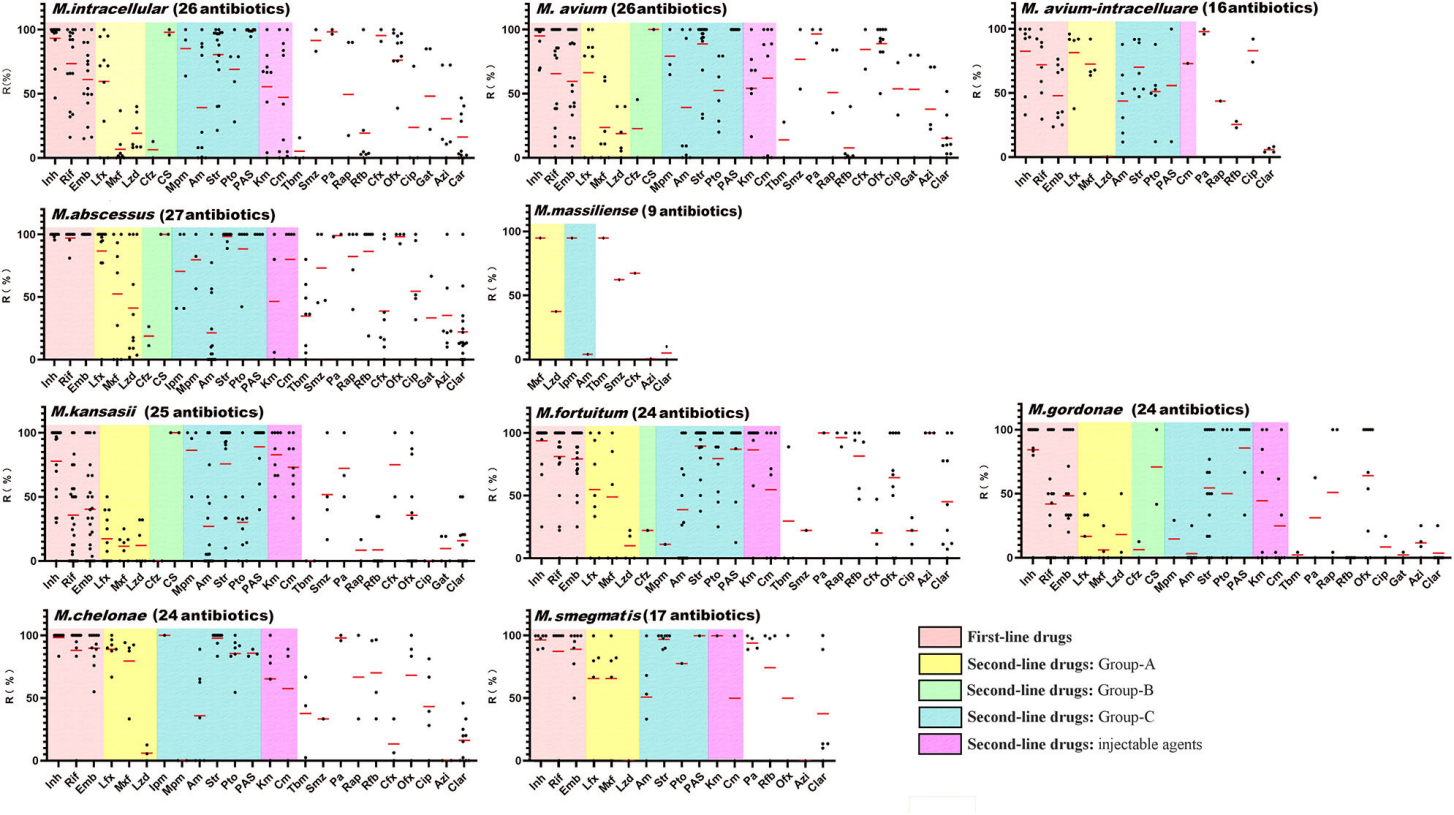
Non-tuberculous mycobacteria isolation rates in mainland China.

In 1979, the first Chinese national TB surveillance program reported that NTM accounted for 4.3% (TB, 717/100,000) of smear-positive clinical cultures. This isolation rate increased to 15.35% (TB, 535/100,000) in the third surveillance (1990), 11.1% (TB, 367/100,000) in the fourth surveillance (2000), and 22.9% (TB, 459/100,000) in the fifth surveillance (2010) (11). As shown in Figure 5, the trends in the prevalence of NTM, as determined in the current study between 2000 and 2010, were consistent with the national monitoring. These data indicated that the prevalence of NTM in China is much higher than that in European countries (0.2–2.9/100,000) (2, 12–14) and in the United States (8.7–13.9/100,000). In addition, the proportion of NTM among mycobacterial isolates in China was higher than that reported in India (6.5%, 2000–2012) (15) and in Iran (10.2%, 1999–2014) (16) but lower than that in Northern India (29%, 2013–2015) (17).

Figure 5 also clearly shows that the changes in NTM prevalence in China are not continuous but rather show periodic fluctuation. Two increasing periods were observed after the two significant decline periods of 2000–2004 and 2010–2015. The NTM isolation rates (/TB suspect and /isolated mycobacteria) between the two decline periods have no statistically significant difference, the same as the NTM isolation rates (/isolated mycobacteria) between the two increasing periods. Although the 95% CIs of the two peaks of the NTM isolation rates (/TB suspects), 6.22–7.20 and 7.32–8.49, do not overlap and indicate statistical significance, the difference is only 1.2%. This analysis indicates that the proportions of NTM in TB are relatively constant in both the increasing and the declining periods. However, the estimated TB incidence (all forms) per 100,000 population in China has gradually dropped from 100 to 85, 72, and 63, down by 37.5% over the same time periods (18). Therefore, although the global NTM prevalence rates are increasing, the NTM infections in the populations in China should be a generally decreasing trend. Moreover, greater awareness of NTM and improved imaging and culture techniques can increase the rates of diagnosis, further implicitly increasing NTM prevalence rates. The decrease of NTM prevalence is perhaps even more so than indicated by the data.

NTM are generally environmental bacteria, so it is reasonable that specific environmental factors relating to soil, water, and temperature can affect the distribution of NTM species and the risk of NTM disease, leading to specific geographic characteristics and time variation. Research showed that MAC was the most common species worldwide, accounting for 34–61% of all NTM isolates (19), followed by *M. gordonae, M. xenopi*, and *M. kansasii*, and *M. fortuitum* was the most common rapid mycobacterial grower (20). Regional analyses showed that *M. kansasii* was the most common species in Slovakia and Poland, *M. xenopi* in Central, Southern, and Western Europe, *M. malmoense* in Northern Europe, and *M. abscessus* complex in North America, Taiwan, Australia, and South Korea (19, 21). At the species group level, MAC was the most prevalent group in China, the same as the rest of the world, although with a less proportion accounting for it (27.46%). However, our results revealed that it is more appropriate to conduct analyses at the species level, which more clearly reflects changes in the prevalence of different species. For example, in China, a shift in the most predominant species from *M. abscessus* to *M. intracellulare* occurred after 2015, with certain differences also noted in the NTM drug resistance spectra.

The geographical distribution of NTM has a general pattern of greater abundance near the coast compared with that inland. For example, the incidence rates of NTM-associated pulmonary disease in Europe were 0.4/100,000 and 0.2/100,000 for coastal and continental regions, respectively (2). Our study revealed a similar distribution pattern in China (11), with an incidence rate of 4.68% in coastal regions compared with 2.9% in western inland regions. In addition to differences in environmental factors, the economic level of the coastal economic zone is higher than that of the inland zone. As a result, the laboratory conditions and the testing capacity of labs in coastal areas are higher than those of labs in inland areas, which is an important contributing factor to differences in prevalence in China. Among the five provinces with the greatest NTM prevalence rates, Guangdong and the four other provinces are more than 10° apart in latitude; thus, it is reasonable that they would show differences in dominant species composition compared with the other provinces. Unexpectedly, the NTM species compositions of three adjoining provinces, Jiangsu, Zhejiang, and Shanghai, were not the same, with the middle province, Shanghai, showing the most divergent composition. Therefore, in addition to environmental factors, we predict that there are also undiscovered factors affecting the distribution of NTM.

Aside from the limited number of drugs to which NTM showed sensitivity, our study exposed two problems with NTM drug sensitivity testing. First, the resistance rates of NTM strains varied greatly between studies. For example, while an NTM species may show 100% resistance to a drug in one study, it could then show 100% sensitivity in another study. Among the 210 drug resistance rates reported for the top 10 NTM species, only 20.48% of rates (43/210) showed a range <25%. While the variations in drug resistance rates were the lowest for *M. abscessus* and *M. gordonae*, only one third of the reported rates were within a variation range of 25%. Therefore, when drug sensitivity testing results are absent or unavailable, physicians should exercise caution when selecting empirical medication for the treatment of NTM infection. Second, we uncovered a lack of drug resistance data for *M. massiliense*.

In conclusion, although the NTM culture and identification methods used in mainland China are still traditional, there is increasing focus on NTM. The current study presents a comprehensive 20-year history of NTM in mainland China, which will further our understanding and aid in the prevention of NTM-associated diseases. The analysis presented here is a valuable reference for clinicians and public health policymakers.

## Author Contributions

LZ and DX independently performed the literature identification and data entry. HL rechecked the data. LZ carried out the data analysis and prepared the first draft of the article. RW conceived the project, analyzed and interpreted the data, and prepared the figures and the manuscript. ZY and KW revised the manuscript. All the authors had full access to all study data and approved the final version of the manuscript for submission.

## Conflict of Interest

The authors declare that the research was conducted in the absence of any commercial or financial relationships that could be construed as a potential conflict of interest.

## References

[1] Gupta RS Lo B Son J. Phylogenomics and comparative genomic studies robustly support division of the genus *Mycobacterium* into an emended genus *Mycobacterium* and four novel genera. Int J Syst Evol Microbiol. (2018) 9:68. 10.3389/fmicb.2018.00067 29497402 PMC5819568

[2] Van Der Werf MJ Kodmon C Katalinic-Jankovic V Kummik T Soini H Richter E. Inventory study of non-tuberculous mycobacteria in the European Union. BMC Infect Dis. (2014) 14:62. 10.1186/1471-2334-14-62 24502462 PMC3922012

[3] Mortaz E Moloudizargari M Varahram M Movassaghi M Garssen J Kazempour Dizagie M. What immunological defects predispose to non-tuberculosis mycobacterial infections? Iran J Allergy Asthma Immunol. (2018) 17:100–9. Available online at: https://ijaai.tums.ac.ir/index.php/ijaai/article/view/1623 29757583

[4] Adjemian J Frankland TB Daida YG Honda JR Olivier KN Zelazny A. Epidemiology of nontuberculous mycobacterial lung disease and tuberculosis, Hawaii, USA. Emerg Infect Dis. (2017) 23:439–47. 10.3201/eid2303.161827 28221128 PMC5382761

[5] Shahraki AH Heidarieh P Bostanabad SZ Khosravi AD Hashemzadeh M Khandan S. Multidrug-resistant tuberculosis may be nontuberculous mycobacteria. Eur J Intern Med. (2015) 26:279–84. 10.1016/j.ejim.2015.03.001 25784643 PMC4414892

[6] Wang L Zhang H Ruan Y Chin DP Xia Y Cheng S. Tuberculosis prevalence in China, 1990–2010; a longitudinal analysis of national survey data. Lancet. (2014) 383:2057–64. 10.1016/S0140-6736(13)62639-2 24650955

[7] Halstrom S Price P Thomson RM. Environmental mycobacteria as a cause of human infection. Int J Mycobacteriol. (2015) 4:81–91. 10.1016/j.ijmyco.2015.03.002 26972876

[8] Wassilew N Hoffmann H Andrejak C Lange C. Pulmonary disease caused by non-tuberculous mycobacteria. Respiration. (2016) 91:386–402. 10.1159/000445906 27207809

[9] Statacorp. Stata Statistical Software 14 edn. College Station, TX: StataCorp LP (2015).

[10] Weiss CH Glassroth J. Pulmonary disease caused by nontuberculous mycobacteria. Expert Rev Respir Med. (2012) 6:597–612. 10.1586/ers.12.58 23234447

[11] Yu X Liu P Liu G Zhao L Hu Y Wei G. The prevalence of non-tuberculous mycobacterial infections in mainland China: systematic review and meta-analysis. J Infect. (2016) 73:558–67. 10.1016/j.jinf.2016.08.020 27717784

[12] Andrejak C Thomsen VO Johansen IS Riis A Benfield TL Duhaut P. Nontuberculous pulmonary mycobacteriosis in Denmark: incidence and prognostic factors. Am J Respir Crit Care Med. (2010) 181:514–21. 10.1164/rccm.200905-0778OC 20007929

[13] Moore JE Kruijshaar ME Ormerod LP Drobniewski F Abubakar I. Increasing reports of non-tuberculous mycobacteria in England, Wales and Northern Ireland, 1995-2006. BMC Public Health. (2010) 10:612. 10.1186/1471-2458-10-612 20950421 PMC2964631

[14] Ringshausen FC Apel RM Bange FC De Roux A Pletz MW Rademacher J. Burden and trends of hospitalisations associated with pulmonary non-tuberculous mycobacterial infections in Germany, 2005-2011. BMC Infect Dis. (2013) 13:231. 10.1186/1471-2334-13-231 23692867 PMC3667050

[15] Raveendran R Oberoi JK Wattal C. Multidrug-resistant pulmonary & extrapulmonary tuberculosis: A 13 years retrospective hospital-based analysis. Ind J Med Res. (2015) 142:575–82. 10.4103/0971-5916.171285 26658593 PMC4743345

[16] Nasiri MJ Dabiri H Darban-Sarokhalil D Hashemi Shahraki A. Prevalence of non-tuberculosis mycobacterial infections among tuberculosis suspects in Iran: systematic review and meta-Analysis. PLoS ONE. (2015) 10:e0129073. 10.1371/journal.pone.0129073 26052701 PMC4460155

[17] Umrao J Singh D Zia A Saxena S Sarsaiya S Singh S. Prevalence and species spectrum of both pulmonary and extrapulmonary nontuberculous mycobacteria isolates at a tertiary care center. Int J Mycobacteriol. (2016) 5:288–93. 10.1016/j.ijmyco.2016.06.008 27847012

[18] World Health Organization WHO TB Burden Estimates. Geneva (2020). Available online at: https://www.who.int/tb/country/data/download/en/

[19] Cowman S Van Ingen J Griffith DE Loebinger MR. Non-tuberculous mycobacterial pulmonary disease. Eur Respir J. 6:210–20. 10.1183/13993003.00250-2019 31221809

[20] Sarro YD Kone B Diarra B Kumar A Kodio O Fofana DB. Simultaneous diagnosis of tuberculous and non-tuberculous mycobacterial diseases: time for a better patient management. Clin Microbiol Infect Dis. (2018) 3:1–8. 10.15761/CMID.1000144 30613797 PMC6319944

[21] Hoefsloot W Van Ingen J Andrejak C Angeby K Bauriaud R Bemer P. The geographic diversity of nontuberculous mycobacteria isolated from pulmonary samples: an NTM-NET collaborative study. Eur Respir J. (2013) 42:1604–13. 10.1183/09031936.00149212 23598956

