# Supplementary material for: Trends in the Prevalence and Antibiotic Resistance of Non-tuberculous Mycobacteria in Mainland China, 2000–2019: Systematic Review and Meta-Analysis
Source: Front Public Health. 2020 Jul 28;8:295. doi: 10.3389/fpubh.2020.00295 (PMC7399041; doi:10.3389/fpubh.2020.00295)
Supplement: Supplementary file 1 [file Data_Sheet_1.PDF]

Supplemental table 1. NTM culture methods used in mainland China in four time periods

Culture Medium	Publication numbers in the four time periods				
	2000-2004	2005-2009	2010-2014	2015-2019	Total
L-J	28	37	62	23	150
BACTEC™ medium	3	2	24	8	37
MacC agar		1	2	20	23
Bact/ALERT 3D	2	3	3	2	10
PNB/TCH		1	2	1	4
Columbia blood agar	2	1		1	4
7H9	1	1		1	3
7H10	2				2
MB/Baet 240		1	1		2
Chromogenic liquid medium of *Mycobacterium*	1		1		2
MTB rapid medium			2		2
Sabouraud agar		1	1		2
Sheep blood agar			2		2
Chocolate plate		1	1		2
7H12B	1				1
BBL™ MGIT™	1				1
China blue agar		1			1

Supplemental table 2. NTM identification methods used in mainland China in four time periods

Identification methods	Identification targets / kits	Publication numbers in the four time periods				
		2000-2004	2005-2009	2010-2014	2015-2019	Total
PNB/TCH culture		25	23	60	17	125
PCR-sequencing	16S rRNA	1	12	24	8	45
	hsp65		0	15	7	22
	rpoB		5	14	3	22
	ITS		5	12	1	18
	RFLP-PCR	1	4	2		7
	Rv0577 (hypothetic)			5		5
	Rv3120 (*tgs1*)			5		5
	IS*1561* (*vapC11*)			4		4
	Rv1510 (hypothetic)			4		4
	Rv1970 (*mpt64*)			4		4
	Rv3877/8 (*eccB2*)			4		4
	sodA		1	3		4
	recA			2		2
	23S rRNA				1	1
	Rv2986 (*leuB*)		1			1
	IS*1245*			1		1
	P6213			1		1
	Rv3349 (*relK*)			1		1
	erm			1		1
	gnd			1		1
	glpL			1		1
	secA			1		1
	dnaA-dnaN		1			1
	NTF-1		1			1
PCR-SSCP	16S rRNA		2			2
PCR-RDBHA	*rpoB*	1	1	2	2	6
Probe	HAIN (CM/AC) kits (Hain Lifescience GmbH, Nehren, Germany)		1	2	2	5
	Mycobacteria Identification Array Kit (CapitalBio, Beijing, China)			6	4	10
	Mycobacterium species identification gene detection kit (Yaneng Biosciences, Shenzhen, China)			1		1<br>0
	BluePoint MycoID (Bio Concept Corporation, Taichung, Taiwan)			1	1	2

	Other kits			2		2
	Microarray chip		3			3
	Whole genome sequencing (WGS)				1	1
	Liquid chromatograph (LC)		1			1
	Mass spectrum (MS)				1	1
Others	High performance liquid chromatography (HPLC)	2	4			6
	Immunohistochemistry			1		1
	PLEX-ID (Abbott)			1		1
	T-SPOT			1	1	2
	Immune colloidal gold technique			1	3	4

Supplemental table 3. Clinical common NTM species in the top 5 NTM isolation provinces and 4 five-year periods.

Rank	Species/complex	Subspecies	2000-2004	2005-2010	2010-2014	2015-2019	Total
1	*M. abscessus*	*abscessus*	494 (24.79%)	1738 (17.05%)	3662 (22.37%)	1981 (20.95%)	7875 (20.72%)
2	*M. intracellulare* (MAC)		60 (3.01%)	1002 (9.83%)	2395 (14.63%)	2258 (23.88%)	5715 (15.03%)
3	*M. avium* (MAC)		146 (7.33%)	399 (3.91%)	891 (5.44%)	816 (8.63%)	2252 (5.92%)
4	*M. avium-intracellulare* (MAC)		95 (4.77%)	1039 (10.19%)	1027 (6.27%)	310 (3.28%)	2471 (6.50%)
5	*M. kansasii*		34 (1.71%)	313 (3.07%)	917 (5.60%)	515 (5.45%)	1779 (4.68%)
6	*M. abscessus*	*massiliense*	0 (0%)	69 (0.68%)	554 (3.38%)	45 (0.48%)	668 (1.76%)
7	*M. fortuitum*		68 (3.41%)	435 (4.27%)	479 (2.93%)	305 (3.23%)	1287 (3.39%)
8	*M. gordonae*		30 (1.51%)	341 (3.35%)	709 (4.33%)	350 (3.70%)	1430 (3.76%)
9	*M. chelonae*		129 (6.47%)	252 (2.47%)	341 (2.08%)	31 (0.33%)	753 (1.98%)
10	*M. smegmatis*		32 (1.61%)	187 (1.83%)	132 (0.81%)	16 (0.17%)	367 (0.97%)
Others			905 (45.41%)	4417 (43.34%)	5265 (32.16%)	2830 (29.92%)	13417 (35.29%)
Total NTM			1993	10192	16372	9457	38014

Rank	Species/complex	Subspecies	Guangdong (65 papers)	Zhejiang (34 papers)	Jiangsu (30 papers)	Shanghai (28 papers)	Beijing (60 papers)
1	*M. abscessus*	*abscessus*	4699	518	78	295	299
2	*M. intracellulare* (MAC)		834	1130	155	336	457
3	*M. avium* (MAC)		504	279	21	108	86
4	*M. avium-intracellulare* (MAC)		1785	35	60	87	1
5	*M. kansasii*		454	196	77	347	211
6	*M. abscessus*	*massiliense*	24	23	0	23	27

7	*M. fortuitum*	679	65	13	62	104
8	*M. gordonae*	489	33	19	100	45
9	*M. chelonae*	444	6	17	46	72
10	*M. smegmatis*	305	8	2	7	3
Total		10217	2293	442	1411	1305

Supplemental table 5. Summary of the average and range of the drug resistance rates (%)of the top 10 NTM species.

Classification	Drugs	*M. abscessus*		MAC			*M. kansasii*	*M. gordonae*	*M. fortuitum*	*M. chelonae*	*M. smegmatis*
		subsp. abscessus	*subsp. massiliense*	*M. avium*	*M. intracellulare*	*M. avium-intracellulare*					
First-line drugs	Inh	99.42 (95.56-100)		95.08 (68.42-100)	93.30 (46.77-100)	82.80 (32.94-100)	77.86 (33.33-100)	84.31 (0-100)	93.70 (25.00-100)	98.33 (83.33-100)	96.67 (88.89-100)
	Rif	97.08 (95.56-100)		65.57 (9.09-100)	73.53 (16.07-100)	72.13 (50-100)	35.68 (0-100)	41.93 (0-100)	81.28 (0-100)	88.02 (0-100)	87.50 (0-100)
	Emb	97.98 (77.78-100)		59.57 (0-100)	61.09 (15.00-100)	48.00 (23.53-76.47)	40.26 (0-100)	48.36 (0-100)	79.34 (0-100)	89.65 (55.00-100)	89.17 (50-100)
Group-A drugs	Lfx	86.79 (0-100)		66.33 (0-100)	59.69 (0-100)	81.78 (37.50-96.00)	17.36 (0-50)	16.67 (0-50)	54.85 (0-100)	88.85 (66.67-100)	65.78 (0-100)
	Mxf	77.17 (27.27-100)		23.61 (0-60)	6.75 (0-36.84)	72.75 (64.00-92.31)	11.52 (0-25.00)	5.96 (0-25.00)	48.82 (0-100)	79.49 (33.33-94.19)	65.78 (0-100)
	Lzd	41.12 (3.57-100)	38.50	18.82 (0-40)	19.22 (8.10-40)	0	12.01 (0-32.05)	18.07 (0-50)	9.96 (0-22.20)	5.99 (0-12.50)	0
Group-B drugs	Cfz	18.76 (11.11-26.40)		22.70 (05-45.40）	6.40 (0-12.80)		0	6.25 (0-12.50)			
	Cs	100		100	97.95 (95.90-100)		100	70.85 (41.70-100)			
Group-C drugs	Imp	70.46 (40.91-100)	97.40							100	
	Mpm	79.70 (82.50-100)		79.21 (64.90-100)	85.27 (63.89-100)		86.39 (50-100)	14.60 (0-29.20)		0	
	Am	22.39 (0-100)	4.30	39.21 (0-100)	39.22 (7.98-100)	43.85 (11.76-88.24)	26.97 (0-100)	3.13 (0-25.00)	38.80 (0-100)	35.81 (0-88.89)	50.97 (0-100)
	Str	98.32 (88.89-100)		88.81 (30.77-100)	80.58 (21.43-100)	70.25 (47.06-92.00)	75.74 (0-100)	53.55 (0-100)	89.51 (37.50-100)	97.70 (83.33-100)	97.08 (88.89-100)
	Pto	88.44 (28.13-100）		52.49 （28.57-79.37)	69.07 (28.13-100）	51.14 (11.76-88.24)	30.10 (0-100)	50 (0-100)	79.59 (25.00-100)	85.52 (54.43-100)	77.78
	PAS	100		100	98.91 (94.59-100)	55.88 (11.76-100)	89.10 (40-100)	85.71 (33.33-100)	87.06 (12.50-100)	85.74 (83.33-88.89)	100
Second-line injectable agents	Km	46.43 (0-100)		54.19 (0-100)	55.46 (0-100)		82.87 (50-100)	44.44 (0-100)	86.55 (57.89-100)	65.22 (0-100)	100
	Cm	80 (0-100)		55.96 (0-100)	55.66 (4.79-100)	73.08	73.06 (33.33-100)	24.88 (0-100)	54.76 (0-100)	57.41 (0-88.89)	50 (0-100)
Others drugs	Tbm	34.79 (0-80)	97.4	13.90 (0-27.8)	5.23 (0-15.70)		0	2.10 (0-4.20)	29.63 (0-88.90)	37.59 (2.34-66.67)	
	Smz	73.16 (45.45-100)	64.10	76.80 (53.60-100)	91.55 (83.10-100)		51.67 (16.67-100)			33.33	
	Pa	99.05 (98.10-100)		96.49 (89.47-100)	98.25 (96.50-100)	98.08 (96.15-100)	72.22 (50-100)	31.25 (0-62.50)		97.92 (95.83-100)	94.17 (88.89-100)

Rap	82.34<br>(80-100)		50.84<br>(0-84.13)	49.35<br>(0-90)	43.75	8.34<br>(0-16.67)	51.05<br>(0-100)	96.30<br>(88.90-100)	66.67<br>(33.33-100)	
Rfb	86.48		7.70<br>(0-40)	19.29<br>(0-100)	25.38<br>(22.76-28.00)	8.66<br>(0-34.62)	0	81.61<br>(47.06-100)	70.03<br>(33.33-95.83)	74.45<br>(0-100)
Cfx	38.70<br>(0-100)		84.55<br>(69.10-100)	95.40<br>(90.80-100)		75.00<br>(50-100)		20.09<br>(0-47.06)	13.19<br>(0-33.33)	
Ofx	98.13<br>(92.50-100)		89.12<br>(50-100)	76.13<br>(38.71-100)		35.61<br>(0-100)	64.13<br>(0-100)	64.38<br>(0-100)	68.06<br>(0-100)	50<br>(0-100)
Cip	54.58<br>(0-100)		53.77<br>(33.33-74.20)	23.83<br>(0-71.50)	83.26<br>(74.20-92.31)	0	8.35<br>(0-16.70)	21.89<br>(11.11-32.35)	43.06<br>(0-81.25)	
Gat	33.34<br>(0-66.67)		53.33<br>(0-80)	48.11<br>(0-85.11)		9.62<br>(0-19.23)	2.10<br>(0-4.20)			
Azi	35.33<br>(21.43-100)	0	37.91<br>(0-70.77)	30.40<br>(0-72.34)			11.55<br>(0-25.00)	100	0	0
Clar	22.06<br>(0-100)	5.20<br>(0-10.40)	15.20<br>(0-51.72)	16.27<br>(0-46.77)	5.59<br>(3.85-8.00)	15.77<br>(0-50)	3.57<br>(0-25.00)	45.07<br>(0-100)	16.15<br>(0-45.83)	37.64<br>(0-88.89)

Note: 1. The abbreviation of drugs were as follow: Inh, isoniazid; Rif, rifampicin; Emb, ethambutol; Lfx, levofloxacin; Mxf, moxifloxacin; Lzd, linezolid; Cfz, clofazimine; Cs, cycloserine; Imp, imipenem; Mpm, meropenem; Am, amikacin; Str, Streptomycin; Pto, protionamide; PAS, aminosalicylic acid; Cm, capreomycin; Km, kanamycin; Tbm, tobramycin; Smz, sulfamethoxazole; Pa, pasinizid; Rap, rifapentine; Rfb, rifabutin; Cfx, cefoxitin; Ofx, ofloxacin; Cip, ciprofloxacin; Gat, gatifloxacin; Azi, azithromycin; Clar, clarithromycin.

2. The drug resistance rates without range were from single publication.

3. The ranges of rates with grey shade are narrower than 25%.
