# Supplementary material for: Trends in the Prevalence and Antibiotic Resistance of Non-tuberculous Mycobacteria in Mainland China, 2000–2019: Systematic Review and Meta-Analysis
Source: Front Public Health. 2020 Jul 28;8:295. doi: 10.3389/fpubh.2020.00295 (PMC7399041; doi:10.3389/fpubh.2020.00295)
Supplement: Supplementary file 2 [file Table_4.pdf]

Supplemental table 4. The breakpoints for resistance determine in the NTM DST in the included publications.

4.1	Breakpoints for *M. abscessus* (μg/mL)			
Drug	Alarmar blue	Absolute concentration	Proportional	Turbidimetry
Inh	5(1,4)<br>8 (1,9)<br>1(2,73)	1,10 (3,73)	0.2(4,76)	–
Rif	1(3,77)<br>8 (1,9)	50,250 (3,73)	40(3,75)	–
Str	5(2,73)<br>10 (2,13)	10,100(3,73)	4(3,75)	–
Emb	4(3,77)<br>10 (1,9)	5,50(3,73)	2(3,75)	–
Am	32 (2,62)<br>64(3,41)	–	–	64(3,106)
Km	4(2,73)<br>64(1,4)	10,100 (1,41)	–	–
Tbm	4 (2,62)<br>8(3,46)	–	–	8(1,22)<br>16(2,76)
Cm	2.5 (2,62)<br>64(1,4)	10,100 (2,72)	40(1,7)	–
Ofx	2 (2,62)<br>4(1,4)	–	2(1,7)	–
Lfx	4 (4,52)<br>8(1,20)	–	–	4(2,36)
Mxf	2 (1,9)<br>4(4,63)	–	–	4(2,36)
Cip	2 (1,9)<br>4(2,24)	–	–	4(2,76)
Gat	4(1,4)	–	–	
Azi	8(3,77)<br>32(1,22)<br>64(1,56)	–	–	8(1,14)<br>32(1,22)
Clar	4 (2,62)<br>8(4,63)<br>32(1,56)	–	–	8(4,112)
PAS	2 (1,9)	1,10 (1,41)	1(2,30)	–
Cs	32(1,4)<br>50 (1,9)	–	–	–
Rap	2(2,57)	–	–	–
Rfb	2 (5,90)	–	–	–
Cfx	64 (2,62)<br>128(3,59)	–	–	128(4,112)
Lzd	16 (2,62)<br>32(4,63)	–	–	32(3,92)
Mpm	16(1,9)<br>32(2,57)	–	–	8(1,40)
Pa	–	–	–	–
Cfz	0.25(1,4)<br>4(1,22)	–	–	–
Imp	32(2,42)	–	–	16(1,20)<br>32(1,22)
Smz	64(1,22)	–	–	64(2,42)
Pto	–	25,100 (1,41)	40(2,68)	–

4.2 Breakpoints for *M. intracellulare* (μg/mL)

Drug	Alamar blue	Absolute concentration	Proportional	Turbidimetry
Inh	1 (2,177)	1,10(4,197)	0.2(7,289)	5(2,57)
Rif	8 (3,548	50,250 (4,197)	40(7,289)	4(3,71)
Str	4 (3,548)<br>10 (1,5)	10,100(4,197)	4(7,289)	10(2,57)
Emb	8 (2,360)<br>10 (1,5)	5,50(4,197)	2(7,289)	8(1,56)<br>4(2,15)
Am	32 (5,741)	–	–	64 (1,56)<br>32(3,16)
Km	10(1,172)	10,100 (3,165)	30(3,106)	–
Tbm	4 (2,177)	–	–	4 (1,1)
Cm	2.5 (2,177)<br>16 (2,376)	10,100 (3,107)	40(4,142)	16 (1,1)
Ofx	2 (2,177)	–	2(4,154)	2(1,1)
Lfx	4 (4,553)	–	–	4(3,71)
Mxf	2 (2,177)<br>4 (3,414)	–	–	4(3,71)
Cip	2 (2,177)	–	–	2(1,1)
Gat	4 (3,548)	–	–	–
Azi	8(3,548)<br>32(1,159)	–	–	32(1,56)<br>512(1,14)
Clar	4 (2,177)<br>32(4,573	–	–	32(2,70)
PAS	2 (2,177)	1,10 (2,75)	1(4,178)	–
Cs	50 (2,177)	–	–	–
Rap	–	–	–	–
Rfb	2 (2,177)<br>4 (2,376)	–	–	4(1,14)
Cfx	64 (2,177)	–	–	
Lzd	16 (3,215)<br>32 (2,376)	–	–	32(2,70)
Mpm	16(2,208)	–	–	
Pa	–	–	–	
Cfz	–	–	–	
Smz	–	–	–	64 (1,56)
Pto	–	25,100 (3,135)	40(3,158)	–

4.3 Breakpoints for *M. avium* (µg/mL)

Drug	Alamar blue	Absolute concentration	Proportional	Turbidimetry
Inh	1(2,100)	1,10 (5,200)	0.2(7,88)	–
Rif	5(2,100)<br>8(1,65)	50,250 (5,200)	40(7,88)	8(1,65)
Str	10(2,100)<br>4(1,65)	10,100 (5,200)	4(7,88)	4(1,65)
Emb	10(2,100)	5,50(5,200)	2(7,88)	8(1,65)
Am	64(2,100)<br>32(1,65)	–	–	32(1,65)
Km	10(1,97)	10,100(4,191)	30(2,22)	–
Tbm	8(2,100)	–	–	–
Cm	2.5(2,100)<br>16(1,65)	10,100(3,74)	40(3,38)	16(1,65)
Ofx	2(2,100)	–	2(4,64)	–
Lfx	4(1,65)<br>8(2,100)	–	–	4(1,65)
Mxf	4(4,175)	–	–	4(2,78)
Cip	4(2,100)	–	–	–
Gat	4(1,65)	–	–	4(1,65)
Azi	8(2,162)<br>32(1,63)	–	–	8(1,65)<br>512(1,13)
Clar	32(2,162)<br>16(2,73)	–	–	32(2,78)
PAS	2(1,3)	1,10(5,200)	1(3,15)	–
Cs	50(1,3)	–	–	–
Rap	2(1,97)	–	–	–
Rfb	2(2,100)<br>4(2,162)	–	–	4(1,65)
Cfx	128(2,100)	–	–	–
Lzd	32(4,175)	–	–	32(2,78)
Mpm	16(2,119)	–	–	–
Pa	0.25(1,97)	–	–	–
Cfz	32(1,97)	–	–	–
Smz	–	–	–	–
Pto	–	25,100(4,140)	40(2,13)	–

4.4 Breakpoints for *M. kansasii* (μg/mL)

Drug	Alamar blue	Absolute concentration	Proportional	Turbidimetry
Inh	1(2,8)	1,10(6,47)	0.2(12,193)	5(2,49)
Rif	1(2,7)<br>5(1,6)	50,250 (6,47)	40(12,193)	1(2,25)<br>2(1,25)<br>32(1,78)
Str	10(2,8)	10,100(6,47)	4(12,193)	10(2,49)
Emb	4(3,85)<br>10(1,6)	5,50(6,47)	2(12,193)	4(2,25)<br>8(1,25)<br>32(1,78)
Am	32(3,85)<br>64(1,6)	–	–	32(3,103)<br>64(1,25)
Km	10(1,2)	10,100 (5,39)	30(3,13)	–
Tbm	8(2,8)	–	–	–
Cm	2.5(2,8)	10,100 (3,36)	40(3,54)	–
Ofx	2(2,8)	–	2(4,19)	–
Lfx	8(2,8)	–	–	2(1,1)<br>4(2,49)<br>16(1,78)
Mxf	2(1,78)<br>64(1,6)	–	–	2(2,25)<br>4(2,103)
Cip	4(2,8)	–	–	
Gat	4(2,4)	–	–	4(2,102)
Clar	8(2,8)<br>16(2,83)	–	–	32(1,25)<br>16(2,25)<br>128(1,78)
PAS	2(2,8)	1,10 (4,38)	1(7,129)	–
Cs	50(2,8)	–	–	–
Rap	2(1,2)	–	–	–
Rfb	1(1,78)<br>2(2,8)	–	–	4(2,49)<br>32(1,78)
Cfx	128(2,8)	–	–	–
Lzd	16(3,85)<br>32(1,6)	–	–	16(2,25)<br>32(1,25)<br>128(1,78)
Mpm	16(1,45)<br>32(2,8)	–	–	–
Pa	–	–	–	–
Cfz	–	–	–	–
Smz	–	–	–	–
Pto	–	25,100(2,11)	40(7,63)	64(1,25)

4.5	Breakpoints for *M. gordonae* (μg/mL)			
Drug	Alamar blue	Absolute concentration	Proportional	Turbidimetry
Inh	5(1,2)	1,10(5,19)	0.2(8,36)	0.2(8,36)
Rif	1(1,2)	50,250(5,19)	40(8,36)	40(8,36)
Str	1(1,2)	10,100(5,19)	4(8,36)	4(8,36)
Emb	4(1,2)	5,50(5,19)	2(8,36)	2(8,36)
Am	32(1,2)	–	–	–
Km	32(1,2)	10,100(4,16)	30(4,22)	30(4,22)
Tbm	–	–	–	–
Cm	32(1,2)	10,100(2,7)	40(2,15)	40(2,15)
Ofx	4(1,2)	–	2(6,27)	2(6,27)
Lfx	4(1,2)	–	–	–
Mxf	4(1,2)	–	–	–
Cip	2(1,2)	–	–	–
Gat	4(1,2)	–	–	–
Azi	4(1,23)<br>32(1,2)	–	–	–
Clar	0.5(1,23)<br>32(1,2)	–	–	–
PAS	–	1,10 (4,13)	1(3,20)	1(3,20)
Cs	–	–	–	–
Rap	–	–	–	–
Rfb	–	–	–	–
Cfx	–	–	128(1,9)	–
Lzd	–	–	32(1,9)	–
Mpm	–	–	–	–
Pa	–	–	–	–
Pto	–	25,100(5,59)	40(7,74)	40(3,11)

4.6 Breakpoints for *M. fortuitum* (μg/mL)

Drug	Alamar blue	Absolute concentration	Proportional	Turbidimetry
Inh	1(2,26)	1,10(9,82)	0.2(13,112)	–
Rif	1(2,26)	50,250(9,82)	40(13,112)	–
Str	5(2,26)	10,100(9,82)	4(13,112)	–
Emb	4(2,26)	5,50(9,82)	2(13,112)	–
Am	64(3,17)	–	–	64(1,9)
Km	4(2,26)	10,100(5,33)	30(4,46)	–
Tbm	8(2,26)	–	–	16(1,9)
Cm	–	10,100(5,27)	40(3,9)	–
Ofx	–	–	2(4,15)	–
Lfx	4(2,10)<br>8(1,17)	–	–	–
Mxf	4(3,27)	–	–	–
Cip	4(2,26)	–	–	4(1,9)
Azi	8(2,23)<br>256(1,9)	–	–	
Clar	8(2,18)<br>32(2,18)	–	–	8(1,9)
PAS	–	1,10(7,70)	1(8,75)	–
Rap	–	–	–	–
Rfb	2(2,26)	–	–	–
Cfx	128(327)	–	–	128(1,9)
Lzd	32(2,10)	–	–	32(1,9)
Pto	–	25,100(5,59)	40(7,74)	–

4.7	Breakpoints for *M. chelonae* (μg/mL)			
Drug	Alamar blue	Absolute concentration	Proportional	Turbidimetry
Inh	–	1,10 (5,134)	0.2(5,139)	–
Rif	–	50,250(5,134)	40(5,139)	–
Str	–	10,100(5,134)	4(5,139)	–
Emb	–	5,50(5,134)	2(5,139)	–
Am	–	–	–	64(2,148)
Km	–	10,100(1,9)	30(2,26)	–
Tbm	–	–	–	16(2,148)
Cm	–	10,100(2,29)	40(1,6)	–
Ofx	–	–	2(1,6)	–
Lfx	–	–	–	–
Mxf	–	–	–	–
Cip	–	–	–	4(2,148)
Azi	–	–	–	–
Clar	–	–	–	8(1,128)
PAS	–	1,10(2,88)	1(3,112)	–
Rap	–	–	–	–
Rfb	–	–	–	–
Cfx	–	–	–	128(2,148)
Lzd	–	–	–	32(2,148)
Mpm	16(2,4)	–	–	–
Pa	–	–	–	–
Imp	–	–	–	16(1,20)
Smz	–	–	–	64(1,20)
Pto	–	25,100 (2,99)	40(5,139)	–

4.8	Breakpoints for *M. massiliense* (µg/mL)	
Drug	Alamar blue	Turbidimetry
Am	32 (2,18)	64(1,9)
Tbm	–	16(1,9)
Azi	–	–
Clar	–	8(1,9)
Cs	–	–
Lzd	–	32(1,9)
Cfz	–	–
Imp	–	–
Smz	–	–

4.9	Breakpoints for *M. avium-intracellulare* (µg/mL)	
Drug	Absolute concentration	Proportional
Inh	1,10 (3,93)	0.2(5,198)
Rif	20,250(3,93)	40(5,198)
Str	10,100 (3,93)	4(5,198)
Emb	5,50 (3,93)	2(5,198)
Am	–	–
Cm	10,100 (1,26)	–
Lfx	–	–
Mxf	–	–
Cip	–	–
Clar	–	–
PAS	–	1(2,139)
Rap	–	–
Rfb	–	–
Lzd	–	–
Pa	–	–
Pto	25,100 (1,50)	40(5,198)

4.10	Breakpoints for *M. smegmatis* (μg/mL)		
Drug	Absolute concentration	Proportional	Turbidimetry
Inh	1,10(3,34)	0.2(4,57)	–
Rif	50,250(3,34)	40(4,57)	–
Str	10,100 (3,34)	4(4,57)	–
Emb	5,50(3,34)	2(4,57)	–
Am	–	–	64(1,1)
Km	–	30(1,2)	–
Cm	–	40(2,3)	–
Ofx	–	–	–
Lfx	–	–	–
Mxf	–	–	4(1,1)
Azi	–	–	–
Clar	–	–	8(1,1)
PAS	1,10(1,22)	1(2,47)	–
Rfb	–	–	–
Lzd	–	–	32(1,1)
Pa	–	1 (1,9)	–
Pto	25,100 (2,99)	40(5,139)	–

Note: 1. Concentration (number of publication, number of strains tested).

2. – the breakpoint has not been clearly listed in the publications, or only noted as: referring to the breakpoints of the species in same subgroup (complex, rapid- and slow-growth *Mycobacterium*).
